# Fluoride distribution in selected foodstuffs from Nakuru County, Kenya, and the risk factors for its human overexposure

**DOI:** 10.1038/s41598-023-41601-8

**Published:** 2023-09-15

**Authors:** Delphine Nelima, Enos W. Wambu, John L. Kituyi

**Affiliations:** Department of Chemistry, School of Science, University of Eldoret, P.O. BOX, Eldoret, 1125-30100 Kenya

**Keywords:** Environmental chemistry, Environmental sciences, Chemistry, Risk factors

## Abstract

Critical data on the impacts of fluoride (F) in food systems along the Eastern Africa Rift Valley System (EARS) is needed for public health risk assessment and for the development of strategies for ameliorating its deleterious effects among the affected communities. Long-term F overexposure causes dental and skeletal fluorosis, and leads to neurotoxicity, which impacts several important body functions. Investigating F exposure pathways is of essence to inform and safeguard public health of the affected communities. The current study assessed the F levels in potatoes (*Solanum tuberosum L.*), beans (*Phaseolus vulgaris L.*) and garden peas (*Possum sativa*) from Nakuru County, Kenya, by potentiometric analysis using F ion-selective electrodes. It then evaluated the risk factors for excessive human exposure to F through contaminated foodstuffs. The mean F levels in the potatoes (8.50 ± 4.70 mg/kg), beans (8.02 ± 4.12 mg/kg) and peas (4.99 ± 1.25 mg/kg) exceeded recommended dietary allowances (RDA) level of 4 mg/kg endorsed by US Institute of Medicine for the different categories of people. The F distribution trends in beans and potatoes reflected the environmental patterns of F contamination of the study area but the spatial extent Fin the peas indicated existence of partial resistance of the pea plants to environmental F uptake. The results indicated that both the beans and the potatoes were more liable to accumulating greater amounts of F from the environment than garden peas and that all the three foodstuffs contained high F levels that posed greater risk of F overexposure and its deleterious impacts among the young children, male populations, and in people of greater body weight and high physical activity levels.

## Introduction

Prolonged fluoride (F) ingestion through foods and water causes adverse health effects in humans, animals and plants. The negative physiological effects of F in humans include dental and skeletal fluorosis, and a myriad of other harmful non-skeletal symptoms that range from mild disturbances in the gut caused by fluoride-induced hyperacidity to severe physiological disorders and toxicities. Dental and skeletal fluorosis develop when excessive fluoride stimulate osteoblastic activity leading to delayed mineralization of bone tissue during its development and resulting in malformed porous and brittle bone structure^1^.

The most common non-skeletal fluorosis appears to spring from the F interference in dietary calcium assimilation. A normal adult human requires approximately 450 mg of daily dietary calcium. Ca is a vital element to the body and it is required for maintaining total health, ensuring strong bones and teeth, maintaining the integrity of skeletal muscles, controlling nerve excitability and aiding in blood clotting^2^. It plays vital roles in spermatogenesis; and in sperm motility, capacitation, acrosome reaction and fertilization^3^. F can precipitae soluble Ca both in the gut and in the blood and lessen its availability to the body inducing its pseudo-deficiency. When blood Ca is too low, the body restores its balance by solubilizing bones’ Ca deposits and vice versa in a process controlled by secretions from the parathyroid glands.

So, F toxicity has been linked to osteoporosis of bones^4^; muscle wasting in animals^5^ and other complications related to carotid artery atherosclerosis^6^ and nephrolithiasis (urinary stones disease)^7^; neurotoxicity manifesting as intelligence suppression^8–14^ and emotional imbalance in children^15^; reproductive^16,17^ and embryo toxicity^18^; and thyroid dysfunction^19^ but even though the association between F toxicity and different kinds of cancers is subject of frequent discuss^20,21^, the causative role of F has not been established. In general, dental and skeletal fluorosis occur in communities depending on drinking water with at least 1.5 and about 3.0 mg/kg F content, respectively. One study reported F neurotoxic effects in 3–4 year-old infants at 0.34 mg daily F exposure^13^ but the most subtle neurological F effects have been reported for threshold drinking water F levels of 1.5 mg/L^8–11,14,15^ and some studies have indicated that structural soft-tissue toxic effects of F may appear at higher threshold drinking water F levels of at least 2.0 mg/L^6,7^

The Eastern African Rift Valley System (EARS) is one of the regions of the world where highest F concentrations of 2800 mg/L have been reported in natural waters^22^. The same has recently been reviewed and discussed extensively in the literature^23^. So, high F levels exceeding permissible standards of 1.5 mg/L for drinking water^24,25^ and reference dose (RfD) of 0.06 mg/kg/day for non-liquid foodstuffs^26^ have been reported^27–29^ among these areas. This has heightened food safety concerns prompting several investigations focused on evaluating the F content and safety for the key foodstuffs from the region^30,31^. Rizzu et al.^31^, for example, assessed the F levels in maize (Zea mays L.), tomato (Lycopersicon esculentum Mill.), and kale (Brassica sp. pl.) along the EARS around Arusha in Tanzania and reported high fluoride contents of 8.0–14.2 mg/kg for the foodstuffs. Despite the ongoing efforts, the F levels in other key staple foodstuffs including potatoes (*Solanum tuberosum L.*), beans (*Phaseolus vulgaris L.*) and garden peas (*Possum sativa*) and the associated risk factors related to consumption of F-contaminated foodstuffs among the most affected communities along the EARS remains unclear. This data is necessary for improving the understanding of the fluoride problem along the EARS high-fluoride belt, and for informing the strategies to deal with fluoride overexposure and ameliorate its deleterious effects among the affected communities.

The current study investigated the F content in Irish potatoes (*Solanum tuberosum L.*), beans (*Phaseolus vulgaris L.*) and peas (*Possum sativa*) from Nakuru County, Kenya, and also assessed the risk of F overexposure among the affected communities. It is anticipated that the results of the current work will contribute to improving knowledge of the local F problem and contribute to the ongoing global efforts to develop credible working solutions to the harmful effects of F overexposure among the communities living in high F regions of the world.

## Material and methods

### Study area

**Figure 1:**
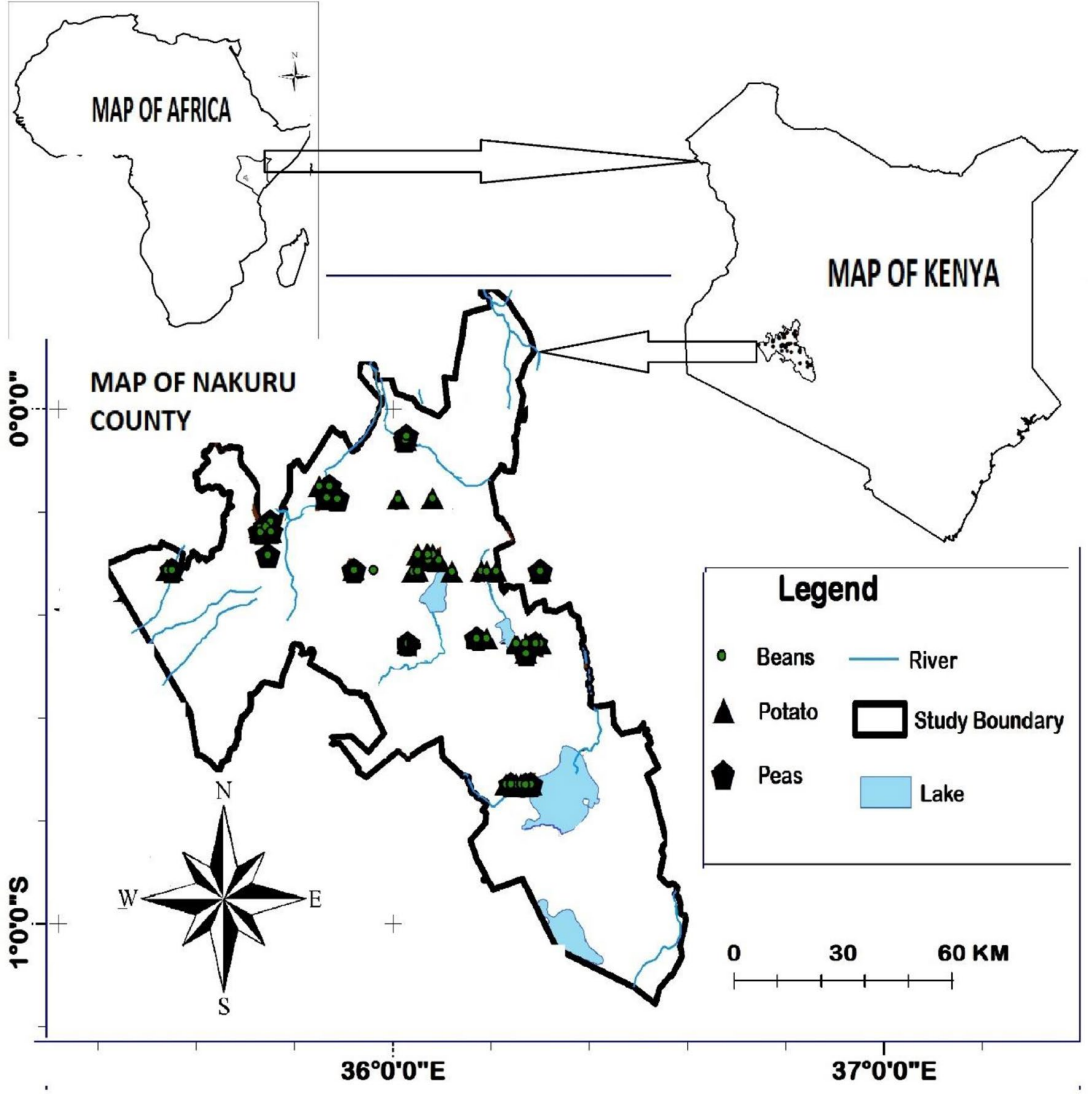
Map of the study area in Nakuru County relative to the map of Kenya and map of Africa.

Figure 1 shows the map of the study area in relation to the map of Kenya and the map of Africa. The area, which comprises Nakuru County of Kenya is about 7500 km^2^ and it lies across latitudes 0º13′ S and 1º10′ S and longitudes 35º28′ E and 35º36′ E. It is divided into 9 administrative sub-counties including: Naivasha, Gilgil, Nakuru East, Nakuru West, Njoro, Nakuru North, Molo, and Rongai, which were the main focus of the current study.

The landscape is composed of a volcanic terrain^32^ and the climate is characterized by a bimodal annual rainfall pattern consisting of short rains in the months of October–December and long rains during the period of March–June^33^. The amount of annual rainfall decreases from over 1500 mm in parts of Njoro and Molo highlands at 2400 m altitude to less than 500 mm in Rongai, Naivasha, Gilgil, Nakuru East and Nakuru West sub-counties at the rift valley floor at about 1600 m elevation. The temperatures range from 12 to 30 °C with the coolest months being June–July and the hottest being December–March.

The varied climatic conditions make the Nakuru County to be one of the mainstay agricultural hubs in Kenya. With a population of close to 2.5 million people comprising 36% urban residents^34^, most of the foods produced are consumed locally with a surplus for external markets and processing.

Children under the age of 13 years, who are at highest risk of the adverse effects of F overdose^35^, make up to 38.2% of the total population of the area^32^.

### Sample collection

Permissions or licenses were obtained from the relevant authorities as required. Field sampling was conducted during the months of August and September in 2019. A total of 165 samples consisting 75 Irish potatoes samples, 54 beans samples and 43 peas samples were collected directly from farms, where they are grown. The samples were handled and used in the present work in accordance with national and international guidelines^36,37^. The sample collection, which was done by random sampling, was carried out from sample points selected to cover the entire study area according to the criterion depicted in Table 1^38^. The raw samples were collected directly into dry polythene *zip lock* bags and transported to the laboratory for processing and testing.

**Table 1:** Distribution of sample collection points by the target sub-counties.

Sub-counties	Foodstuffs			
	Potatoes (*n*)	Beans (*n*)	Peas (*n*)	Total (*N*)
Molo	10	5	8	23
Njoro	10	7	9	26
Rongai	9	6	6	21
Nakuru N	11	5	6	22
Nakuru W	10	4	4	18
Nakuru E	10	4	3	17
Gilgil	7	8	3	18
Naivasha	8	8	4	20
Overall	75	47	43	165

### Sample preparation

At the laboratory, the samples were washed with excess doubly-deionized water (DDW) to remove soils and other impurities. The potatoes were sliced into cubes of about 1.5 cm dimensions and dried on a ULM-400 constant-temperature oven (Memmert, Germany) at 110 °C for 24 h. The peas and beans samples were dried in the same way without further treatment. The dry samples were pulverized to a fine powder using an SBG-301 Blender/Grinder (ASL Ltd, Kenya) and preserved in air-tight sample bottles for the subsequent tests.

### Extraction of fluoride from the samples

A portion of 1.25 g of each of the powdered samples (beans, potatoes and peas) was digested with 10 mL of 6 M NaOH on a BKD-20F Kjeldahl Digestion Furnace (Biobase, China) until the samples dissolved in the base. The solution was allowed to cool to room temperature and neutralized by careful addition of 3 M H_2_SO_4_ to pH 7.1 ± 0.1 before it was transferred into a 50-mL volumetric flask and made up to the 50-mL volumetric mark with DDW.

### Fluoride determination using ion-selective electrodes

Exactly 10.0 mL of each of the sample solutions was mixed with an equal volume of total ionic strength adjusting buffer (TISAB-III) solution^39^ and the F concentration measured using a Thermo Fisher Scientific 9609BNWP pH/Ion Meter (STAR) with a Thermo Fisher Fluoride ion selective electrode (Thermo Fischer Scientific, UK)^38^. All the experiments were conducted in triplicate.

### Statistical analysis

The results were analyzed using IBM SPSS Statistics Software by determining the mean and standard deviations on replicate data and causal relationships determined by the Analysis of Variance (ANOVA) to determine whether there were any significant differences (p ≤ 0.05) between the concentrations of fluoride in peas, beans and potatoes from different regions of the study area. The paired t-test was then applied to determine significant difference at p ≤ 0.05 confidence level in fluoride level between peas, beans and potatoes and to compare means from different regions.

The results were used to calculate the daily F intake (DFI) in mg/kg/day for individual foodstuffs and to compute the estimated average daily F dosage (EADD) in mg/kg/day for the households.

### Determination of the risk of fluoride overexposure

#### Daily food energy contribution (FEC) by the foodstuffs

The average daily household food intake (*ADFC*_*i*_) in kg for the particular *i*^*th*^ foodstuff was determined from food conservation and dietary diversification data and from the food consumption recall data and production survey conducted previously in the study^40,41^. The energy contribution (*FEC*) of the *ith* foodstuff, which is the fractional/ percentage contribution of the particular type of foodstuff to the overall daily energy consumption of an overage individual/household was estimated according to Eq. (1) as:

1 $${FEC}_{i}= \frac{{ADFC}_{i}\times {E}_{i}}{DEI}\times 100,$$

where *E*_*i*_ is the calorific value (kcal/kg) of the *ith* foodstuff and *DEI* (kcal/day) is the total daily energy intake from all foodstuffs in the person’s or household daily menu. The daily energy intake (*DEI*) data for human energy requirements by age categories, body weights (BW) and physical activity levels (PAL) have been published and was obtained for the current calculations from a joint report of Food and Agriculture Organization (FAO), World Health Organization (WHO) and the United Nations University (UNU)^42^.

#### Daily fluoride intake, DFI, (mg/kg/day) from the foodstuffs

The daily fluoride intake, *DFI*, (mg/kg/day) through consumption of the particular *ith* foodstuff was then calculated from Eq. (2)^30,31^ based on the determined values of *FEC*_*i*_ and *DEI* as:

2 $${DFI}_{i}=\frac{{[F]}_{i}\times {DEI}\times {FEC}_{i}\times AF}{{E}_{i}},$$

where, *[F]*_*i*_ is the F content of *ith* foodstuff (mg/kg); *AF* is the F absorption factor estimated at 60% for most non-liquid foodstuffs^43^; whereas *E*_*i*_ is the dietary calorific value (kcal/kg) of the *ith* foodstuff. The F content of the foodstuff in mg/kg, *[F]*_*i*_, was determined experimentally for beans, peas and potatoes in the current work and the values for the other foodstuffs obtained from the relevant literature^28,44^.

The US Centers for Disease Control and Prevention (CDC) through the National Center for Health Statistics publishes Data Tables of Weight-for-age Charts that provide information on the distribution of body weights (BW) by human age categories^45^. The daily human energy requirements used for estimating *FEC*_*i*_ and *DFI*_*i*_ according to Eqs. (1) and (2) above was obtained from the joint data report of FAO/WHO/UNU^42^ by adopting the 50^th^ percentile weight in each age category as the representative weight for the particular age.

#### Estimated average daily fluoride dosage (EADD_c_)

The estimated daily fluoride dosage, *EADD*_*c*_^30^ for *n* number of food items in the average daily household menus was then computed by adding together the daily fluoride intake, *DFI*, (mg/kg/day) for individual foodstuffs according to Eq. (3) as:

3 $${EADD}_{c}=\sum_{i}^{n}{DFI}_{i}.$$

The results were compared with the recommended dietary allowance (RDA) published in the literature for the different categories of people^46^.

## Results

### Spatial distribution of the fluoride levels in peas, beans and potatoes

The results of the analyses of F levels of the three foodstuffs by the sub-counties are presented in Table 2. The F content of the foodstuffs was found to follow the order:

4 $${[F]}_{peas}\ll {\left[F\right]}_{beans}\approx {\left[F\right]}_{potatoes},$$

where, [F]_*peas*_, [F]_*beans*_, and [F]_*potatoes*_ are the F concentrations (mg/kg) of the respective foodstuffs. The highest mean F levels in peas were recorded in Nakuru East (5.64 ± 0.70 mg/kg) whereas the lowest Naivasha (3.51 ± 1.73 mg/kg) sub-counties. However, the highest mean F contents of the beans (14.50 ± 4.34) and of the potatoes (12.50 ± 5.53 mg/kg) were found among the samples collected from Nakuru North and Molo sub-counties, respectively. The general trends in the overall F levels of the three foodstuffs were as follows:

**Table 2:** Spatial distribution of [F] mg/kg in the three foodstuffs.

Sub County	[F] in Beans (mg/kg)		[F] in Potatoes (mg/kg)		[F] in Peas (mg/kg)	
	Range	Mean ± Stdev	Range	Mean ± Stdev	Range	Mean ± Stdev
Gilgil	2.97–8.92	5.81 ± 2.21	3.47–7.89	5.43 ± 1.52	3.86–4.18	3.97 ± 0.18
Molo	4.9–9.6	6.67 ± 1.60	3.99–18.88	12.50 ± 5.53	3.62–6.64	5.43 ± 1.06
Naivasha	2.15–5.66	4.27 ± 1.07	2.75–6.56	4.57 ± 1.63	1.94–5.28	3.51 ± 1.73
Nakuru East	6.14–10.59	8.24 ± 1.84	4.86–10.72	7.05 ± 1.95	4.88–6.35	5.64 ± 0.70
Nakuru North	10.51–21.44	14.50 ± 4.34	4.8–18.05	10.77 ± 4.81	3.77–6.89	5.37 ± 1.16
Nakuru West	8.88–18.2	12.90 ± 3.97	3.31–12.79	9.55 ± 2.58	3.42–7.12	5.41 ± 1.63
Njoro	3.74–5.99	4.98 ± 0.84	3.82–12.9	7.10 ± 3.10	3.57–5.94	4.89 ± 0.84
Rongai	10.59–17.47	14.21 ± 2.85	3.72–6.58	5.02 ± 0.89	3.57–7.89	4.85 ± 1.59
Overall	2.15–21.44	8.50 ± 4.70	2.75–18.88	8.02 ± 4.12	7.89	4.99 ± 1.25

$$Beans:\, Nakuru\, North\, >\, Rongai\, > \,Nakuru \,West\, > \,Nakuru \,East$$

$$Potatoes: \,Molo\, > \,Nakuru \,West \,>\, Njoro\,> \,Nakuru \,East$$

$$Peas: \,Nakuru\, East \,\ge\, Molo\, \approx \,Nakuru \,West\, \approx \,Nakuru\, North\, \ge \,Njoro$$

High F levels in the three foodstuffs were detected in areas at the rift valley floor, which have been reported to be the fluoride hotspots^47^.

### Statistical analyses

The results of the analysis of the variance (ANOVA) showed that there was no significant difference in the F content of peas samples between the 8 sub-counties *(p* = 0.436*)* of Nakuru County, Kenya. Nonetheless, the F levels of both the beans *(p* = 0.000) and potatoes *(p* = 0.000) were significantly different across the 8 sub-counties studied (*p* = 0.000*)*.

The paired t-test was applied to compare the means of F levels between the peas and the beans, the peas and potatoes, and between the beans and the potatoes. It was found that the mean F levels were significantly different (*p ˂ 0.05*) between the peas and the beans (*p* = 0.033) and between peas and potatoes (*p* = 0.030) but that the means of the F levels between beans and potatoes (*p* = 0.067) were not significantly different from each other.

The paired t-test was then also applied to compare the mean F levels in the peas between Njoro and Naivasha and between Nakuru and Gilgil regions to test if significance differences existed within the same dataset across the regions. It was found that no significant differences existed in the mean F levels in the peas between Njoro and Naivasha regions (*p* = 0.051) but there was strong significance between the mean F levels in peas samples obtained from Nakuru and Gilgil regions (*p* = 0.00).

Then, by applying the paired t-test to compare the mean F levels in the beans and in the potatoes between Njoro and Naivasha and between Nakuru and Gilgil regions (*p* ˂ 0.05), it was found that the mean F levels in the beans were significantly different between Njoro and Naivasha (*p* = 0.000) and also between Nakuru and Gilgil (*p* = 0.020). In the same way, the results confirmed that the mean F levels in the potatoes were significantly different between Njoro and Naivasha (*p* = 0.001).

The foregoing results from application of the paired t-test agreed with the results obtained from the analysis of the variance (ANOVA) in this section.

### Analyses of fluoride risk of overexposure

#### Daily food energy contribution (FEC)

In order to assess the potential of F overexposure through the foodstuffs, household dietary patterns of local communities were analyzed based on food diversification and the food production data for the area in order to identify the most used foodstuffs and estimate the food energy contribution (FEC) of the foodstuff to the daily energy intake as in Eq. (2) and the results depicted in Table 3.

**Table 3:** The main foodstuffs contributing to daily household dietary energy requirement in Nakuru County, Kenya.

Food items	Food energy content	Av. weekly food consumption	Av. daily food consumption	Daily food energy contribution	Food F content	Daily food energy contribution	Refernce
	${E}_{I}$	${AWFC}_{I}$	${ADFC}_{I}$	${E}_{i}\times {ADFC}_{i}$	[F]	${FEC}_{I}$	
	(kcal/kg)	(kg)	(kg)	(kcal)	(mg/kg)	(%)	
Maize (*Zea mays*)	1110	6.89	0.98	1092.56	13.6	27.9	^44^
Irish potatoes (*Solanum tuberosum L.*)	1050	6.5	0.93	975.00	8.2	24.9	Current study
Garden peas (*Possum sativa*)	1200	3.35	0.48	574.29	4.9	14.7	Current study
Carrots (*Daucus carota*)	320	2.8	0.40	128.00		3.3	
Cabbage (*Brassica oleracea capitata*)	1000	2.1	0.30	300.00		7.7	
Onions (*Allium cepa*)	420	2.1	0.30	126.00		3.2	
Sukuma (*Brassica oleracea*)	540	2.1	0.30	162.00	13.3	4.1	^28^
Tomatoes (*Lycopersicum esculentum*)	280	2.1	0.30	84.00	12.9	2.1	^28^
Beans (*Phaseolus vulgaris L.*)	1090	1.5	0.21	233.57	8.7	6.0	Current study
Others (e.g., tea beverage etc.)	608	2.8	0.40	243.20		6.21	
Overall		${\sum }_{i}^{n}\left(AWFC\right)=32.24 \,kg$	${\sum }_{i}^{n}\left(ADFC\right)=4.61 \,kg$	$DEI=\sum_{i}^{n}\left({E}_{i}\times ADF{C}_{i}\right)=3918.61 \,kcal$			

The average household size in Nakuru is 3.0–3.9 persons^34^. It was found that a typical household consumes about 3918.61 kcal of food daily in form of the popular local maize meal known as *ugali* (27.88%), Irish potatoes (24.88%), garden peas (14.66%), and leafy vegetables, especially cabbages and a popular local variety of kales known as *Sukuma wiki*. These foodstuffs, which are depicted in Table 4 contribute approximately 93.79% of the daily household energy needs of the members.

**Table 4:** Calculated EADD values for the main foodstuffs consumed daily by residents in Nakuru County.

Age category (years)	Recommended limits		Upper tolerable limits^46^ (mg/day)	Three foods* (mg/day)		Five foods ** (mg/day)	
	Male (mg/day)	Female (mg/day)		Male	Female	Male	Female
				Mean ± Stdev	Mean ± Stdev	Mean ± Stdev	Mean ± Stdev
1–3	0.7	1.0	1.3	2.00 ± 0.28	1.85 ± 0.27	5.61 ± 0.77	5.17 ± 0.74
4–8	1.0	2.0	2.2	2.76 ± 0.39	2.53 ± 0.36	7.74 ± 1.08	7.09 ± 1.02
9–13	2.0	3.0	10.0	4.10 ± 0.64	3.72 ± 0.47	11.48 ± 1.79	10.42 ± 1.31
14–18	3.0	3.0	10.0	5.66 ± 0.47	4.45 ± 0.10	15.86 ± 1.31	12.47 ± 0.27
≥ 19	4.0	3.0	10.0	5.30 ± 1.05	4.47 ± 0.84	14.87 ± 2.95	12.54 ± 2.36

*Beans, potatoes and garden peas studied in the current work.

**Beans, potatoes and garden peas studied in the current work plus maize, tomatoes and *sukuma wiki* whose from content had been previous studied in the literature.

#### Daily fluoride intake (DFI_i_) and estimated average daily F dosage, EADD values

**Figure 2:**
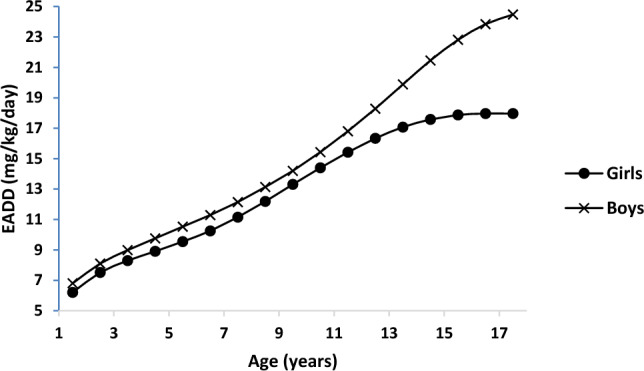
Amounts of dietary F exposure through food for boys and girls aged 1–18 years.

Based on the FEC ratios in Table 4, the daily fluoride intake, DFI, for the food items and the average daily dosage of fluoride (EADD) for the different age groups, gender, body weight (BW), and physical activities level (PAL) were estimated using Eq. (2) and (3) and the results presented in Fig. 2 and in Table 4.

The mean EADD values were 14.87 ± 2.95 and 12.54 ± 2.36 mg/day for males and females above the age of 18 years, respectively, and 5.61 ± 0.77—11.48 ± 1.08 and 5.17 ± 0.74—10.42 ± 1.31 mg/day for male and female children in the age bracket 1–13 years, respectively. These results are in agreement with those of previous researchers who found that the daily F intake by a group of nursing mothers averaged 22.1 mg/day with a range of 9.5–37.2 with cooked food, water and tea contributing 11.7, 4.5 and 5.8 mg of F exposure daily^48^. For all the age categories and in both males and female, the calculated EADD values for the foodstuffs exceeded recommended dietary limits quoted in Table 4^46^.

#### Effect of age and gender

From the results present in Fig. 2, it was observed that F exposure was higher in boys than in girls and that the estimated average daily F dosage (EADD) increased with increasing age of the children from one year of age and it leveled off at about age of 18 and 16 years for the boys and the girls, respectively.

It can be noted that in the age-bracket of 1–12 years, the EADD for boys was about 1.10 times higher than that for the girls at all ages. This ratio increased to about 1.36 times in the adolescent age bracket of 12–18 years, which was consistent with similar results reported elsewhere in the literature. In a previous survey involving 3771 adult males and 4495 adult females on the risks of F-instigated bone fractures among rural communities in China, for example, the authors indicated that the F-instigated bone fractures were 1.35 more prevalent among the male populations than among the corresponding female categories^49^. In an epidemiological survey on the pattern of abnormal urinary fluoride levels among populations with occupational fluoride exposure in Shanghai, it was reported that the prevalence rates of abnormally high urinary fluoride in the men were about 4.46 times greater than in the women. These values collaborate the results of the present work, which have revealed higher tendency of males to suffer greater impact of F than females under the same conditions of exposure.

#### Impact of body weight (BW) and physical activity levels (PAL)

**Figure 3:**
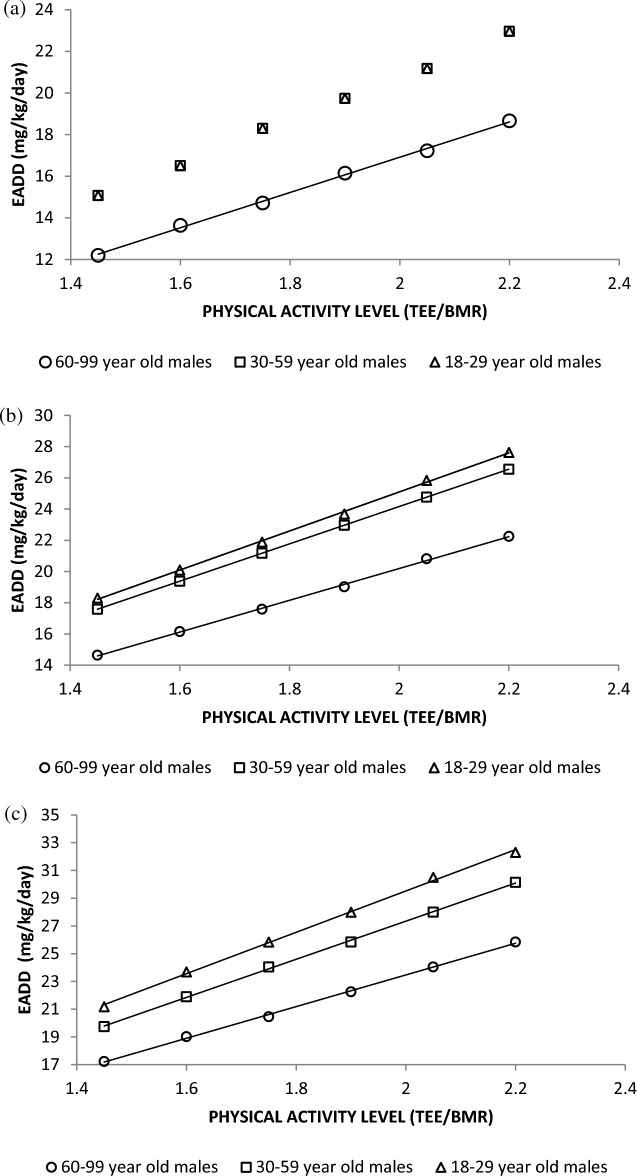
Representative plots of estimated daily F dosage (EADD) for different categories of BW and PAL by gender and by different age groups for: (**a**) 50 kg BW, (**b**) 70 kg BW, and (**c**) 90 kg BW males (*NB: TEE is the total energy expenditure in 24 h and BMR is the basal metabolic rate*).

The levels of EADD were compared for the different categories of BW and PAL by gender and age-groups and the results presented in Fig. 3a–c.

The results showed that the risk of F overexposure through the foodstuffs increased with increasing BW and increasing PAL and that the potential of F overexposure was greater for the young adults (18–29 years) than among the older categories of people. It could be observed that at low BW of 50 kg, the F exposure levels for young men (18–29 years) was similar to those for men of 30–59 years of age but it differed from those for older men above 60 years of age. Figure 3b,c show that the value of EADD was different for the 18–29, 30–59, and for 60-and-above age categories of BW of 70 kg and above.

## Discussion

As depicted in Table 4, the results in the current study showed that peas, beans and Irish potatoes from Nakuru County contained high levels of F above the recommended dietary allowance (RDA) for the different groups of people^46^. However, whereas high F was recorded in all the three foodstuffs obtained from all the regions of the current study area, the F levels in garden peas were much lower when compared to those in the beans and the potatoes. Furthermore, the results of the analysis of the variance (AVOVA), showed that F levels in the beans and in the potatoes were not statistically different even though both of them were significantly different between the regions of the current study area. The F content in the peas was, however, not statistically different between the regions despite the heterogeneity in spatial distribution of fluoride in the environment^50^.

High fluoride occurrence in beans and potatoes, depicted in Table 2, were consistent with the prevailing F distribution trends in environment^47^ and it was heightened within the fluoride pollution hotspots located at the hotter and drier areas of rift valley floor including Nakuru North, Nakuru West, Molo, Rongai and Nakuru East sub-counties^47^. This was attributed to uptake and accumulation of F in the bean^51^ and potato^52^ plants from F-enriched soils resulting from long-term sedimentation of high-fluoride debris by agents of erosion and deposition (accentuated by evaporative concentration due the hotter climate of the low altitude regions). This showed that people relying on these foods, derived from crops cultivated in these areas, were at greater potential of fluoride overexposure.

The differences in the mean F levels of the three foodstuffs (Table 2) produced within similar conditions can be explained, in part, on the variations in the local soil and soil–water F conditions and on dissimilarities in ecological conditions. Other than ecological conditions affecting the F accumulation of the respective crops, the prevailing land use forms and practices, and other human activities can influence the availability of soil–water F content to varying degrees^53^. Also, differences in the local geology and volcanic terrains resulting from presence of active geothermal activities^54^ can occur over limit geographical space^55^ causing minor variations in rock solubilization processes impacting soil geochemistry and the net spatial F concentration in the soil–water F^56^. This, in turn, controls the soil-F interactions with plant species^57^. The high F content in the foodstuffs obtained from higher altitude areas of Molo and Njoro regions could, therefore, be linked to the presence of high-F volcanic landscapes^58^ and to the resulting F soils enrichment due to the ascent of hydrothermal fluids of active geothermal activities occurring in parts of these areas^59^.

The diverse genotypic traits of plant species can also affect the extent to which the plant species absorb^60^ and tolerate F levels in the soils^61^. The low F concentrations in the pea’s samples across the regions of Nakuru County when compared to the F levels of the beans and the potatoes samples, indicated that F uptake by the pea’s plants was not influenced by the ecological, climatological and geological F conditions of the regions. It showed that the pea plant was somewhat resistant to F uptake from the environment than both the beans and the Irish potatoes, which can have implications on safe food production.

The results from the analysis of the risk of F exposure from the foodstuffs given in Fig. 2 and in Table 4 revealed that the male populations of the resident communities living in high F belts are at the greater risks of F overexposure compared to the corresponding female populations under matching conditions. It showed that under similar conditions, the male populations were prone to ingesting greater dietary amounts of F exposing themselves to greater prevalence and more severe F impacts than their female counterparts.

A summary of similar discrepancies in the effects of F overexposure between the male and female human populations reported in the literature from around the world are presented in Table 5.

**Table 5:** Differential impact of fluoride on the male and female populations reported in the literature all over the world.

Fluoride adverse health effect	Place of study	Sample size (n)	Female Index (*I*_*f*_)	Male index (*I*_*m*_)	Units	*I*_*m*_*/I*_*f*_	Literature source	Age bracket (years)	Age category
Dental fluorosis prevalence (< 1.5 mg/L water F)	Villages in Vadodara district, Gujarat, India, with < 1.5 mg/L F water	244	4.58	6.67	%	1.46	^62^	0–4	Children
Dental fluorosis prevalence (> 1.5 mg/L water F)	Villages in Vadodara district, Gujarat, India, with > 1.5 mg/L F water	315	7.04	7.55	%	1.07	^62^	0–4	Children
Dental fluorosis prevalence	Ledhupur & Rustampur, Varanasi	218	4.49	4.58	%	1.02	^63^	1–5	Children
Dental fluorosis prevalence	Nawa tehsil, Nagaur district, Rajasthan (India)	60	90	91.67	%	1.02	^64^	4–16	Children
Blood serum/plasma F	Balavenkatapuram, Kalyandurg Mandal, Anantapur, Andhra Pradesh, India	10	0.18	0.51	ppm	2.83	^65^	5–11	Children
Urine F	Ayyagarlapalli, Setturu Mandal, Anantapur District, Andhra Pradesh, India	10	2.14	3.08	ppm	1.44	^65^	5–11	Children
Urine F	Kanukuru, Setturu Mandal, Anantapur District, Andhra Pradesh, India	10	5.12	7.26	ppm	1.42	^65^	5–11	Children
Urine F	Balavenkatapuram, Kalyandurg Mandal, Anantapur, Andhra Pradesh, India	10	4.12	5.4	ppm	1.31	^65^	5–11	Children
Blood serum/plasma F	Ayyagarlapalli, Setturu Mandal, Anantapur District, Andhra Pradesh, India	10	0.36	0.46	ppm	1.28	^65^	5–11	Children
Dental fluorosis prevalence (< 1.5 mg/L water F)	Villages in Vadodara district, Gujarat, India, with < 1.5 mg/L F water	663	36.76	38.01	%	1.03	^62^	5–11	Children
Blood serum/plasma F	Kanukuru, Setturu Mandal, Anantapur District, Andhra Pradesh, India	10	0.64	0.64	ppm	1.00	^65^	5–11	Children
Dental fluorosis prevalence (> 1.5 mg/L water F)	Villages in Vadodara district, Gujarat, India, with > 1.5 mg/L F water	533	63.94	56.67	%	0.89	^62^	5–11	Children
Dental fluorosis prevalence (DMFT scores)	Thailand	271	0.2	0.1	DMFT score	0.50	^66^	5–9	Children
Dental fluorosis prevalence	Libyan Population	6244	62.28	64.27	%	1.03	^67^	6–60	All
Dental fluorosis prevalence	Rural Area, Tianjin, China	709	46.26	53.74	%	1.16	^68^	6–13	Children
Dental fluorosis prevalence	Ledhupur & Rustampur, Varanasi	256	37.19	37.1	%	1.00	^63^	6–10	Children
Dental fluorosis prevalence	Israel-administered Gaza Strip	152	52.6	47.4	%	0.90	^69^	6–8	Children
Dental fluorosis prevalence	Han, China	182	9.33	10.28	%	1.10	^70^	8–15	Adolescents
Dental fluorosis prevalence	Tibetan, China	375	52.47	50.23	%	0.96	^70^	8–15	Adolescents
Dental fluorosis prevalence	Alappuzha district, Kerala, India	1124	39.2	31.3	%	0.80	^71^	10–17	Adolescents
Dental fluorosis prevalence	Thailand	299	0.8	0.4	DMFT scores	0.50	^66^	10–14	Adolescents
Dental fluorosis prevalence	Ledhupur & Rustampur, Varanasi	158	53.94	58.86	%	1.09	^63^	11–15	Adolescents
Dental fluorosis prevalence	Fluorotic rural area, Turkey	293	4.75	8.13	%DMFT scores	1.71	^72^	12–80	All
Dental fluorosis prevalence (< 1.5 mg/L water F)	Villages in Vadodara district, Gujarat, India, with < 1.5 mg/L F water	744	40.2	48.87	%	1.22	^62^	12–24	Adolescents
Dental fluorosis prevalence (> 1.5 mg/L water F)	Villages in Vadodara district, Gujarat, India, with > 1.5 mg/L F water	599	71.83	83.74	%	1.17	^62^	12–24	Adolescents
Blood serum/plasma F	Kanukuru, Setturu Mandal, Anantapur District, Andhra Pradesh, India	8	0.35	0.39	ppm	1.11	^65^	12–18	Adolescents
Blood serum/plasma F	Ayyagarlapalli, Setturu Mandal, Anantapur District, Andhra Pradesh, India	10	0.17	0.18	ppm	1.06	^65^	12–18	Adolescents
Urine F	Kanukuru, Setturu Mandal, Anantapur District, Andhra Pradesh, India	8	6.87	7.18	ppm	1.05	^65^	12–18	Adolescents
Urine F	Ayyagarlapalli, Setturu Mandal, Anantapur District, Andhra Pradesh, India	10	3.58	2.67	ppm	0.75	^65^	12–18	Adolescents
Dental fluorosis prevalence	Schools in Southern India	1025	63.6	65.2	%	1.03	^73^	12–17	Adolescents
Dental fluorosis prevalence	Nalgonda district, Andhra Pradesh, India	775	77	78	%	1.01	^74^	12	Adolescents
Dental fluorosis prevalence	Nalgonda district, Andhra Pradesh, India	778	76.9	78.8	%	1.02	^74^	15	Adolescents
Dental fluorosis prevalence	Ledhupur & Rustampur, Varanasi	95	27.41	54.73	%	2.00	^63^	16–20	Adults
Dental fluorosis prevalence	Nawa tehsil, Nagaur district, Rajasthan (India)	28	81.82	95.93	%	1.17	^64^	17–28	Adults
Urine F	Ayyagarlapalli, Setturu Mandal, Anantapur District, Andhra Pradesh, India	9	2.13	2.55	ppm	1.20	^65^	19–35	Adults
Blood serum/plasma F	Ayyagarlapalli, Setturu Mandal, Anantapur District, Andhra Pradesh, India	9	0.22	0.24	ppm	1.09	^65^	19–35	Adults
Dental fluorosis prevalence (DMFT scores)	Thailand	116	1.2	0.8	DMFT scores	0.67	^66^	20–24	Adults
Dental fluorosis prevalence	Ledhupur & Rustampur, Varanasi	91	22.41	42.83	%	1.91	^63^	21–25	Adults
Dental fluorosis prevalence (DMFT scores)	Thailand	261	0.9	0.6	DMFT scores	0.67	^66^	24–34	Adults
Dental fluorosis prevalence (> 1.5 mg/L water F)	Villages in Vadodara district, Gujarat, India, with > 1.5 mg/L F water	302	60.27	67.3	%	1.12	^62^	25–44	Adults
Dental fluorosis prevalence (< 1.5 mg/L water F)	Villages in Vadodara district, Gujarat, India, with < 1.5 mg/L F water	442	40.62	46.77	%	1.15	^62^	25–44	Adults
Dental fluorosis prevalence (> 1.5 mg/L water F)	Villages in Vadodara district, Gujarat, India, with > 1.5 mg/L F water	480	64.45	72.32	%	1.12	^62^	25–34	Adults
Dental fluorosis prevalence (< 1.5 mg/L water F)	Villages in Vadodara district, Gujarat, India, with < 1.5 mg/L F water	516	42.33	45.37	%	1.07	^62^	25–34	Adults
Dental fluorosis prevalence	Ledhupur & Rustampur, Varanasi	80	16.66	31.25	%	1.88	^63^	26–30	Adults
Dental fluorosis prevalence	Nawa tehsil, Nagaur district, Rajasthan (India)	49	95	96.43	%	1.02	^64^	29–40	Adults
Dental fluorosis prevalence	Ledhupur & Rustampur, Varanasi	299	5.12	16.72	%	3.27	^63^	31	Adults
Dental fluorosis prevalence	Thailand	202	1.5	1.5	DMFT scores	1.00	^66^	35–44	Adults
Blood serum/plasma F	Xinhuai, Sihong County, Jiangsu Province, China	36	0.073	0.077	mg/L	1.05	^75^	36–78	Adults
Skeletal fluorosis prevalence	Wamiao, Sihong County, Jiangsu Province, China	132	36.21	27.03	%	0.75	^75^	36–78	Adults
Dental fluorosis prevalence	Nawa tehsil, Nagaur district, Rajasthan (India)	20	94.44	100		1.06	^64^	40 <	Adults
Dental fluorosis prevalence	Thailand	297	8.1	9.2	DMFT scores	1.14	^66^	45 <	Adults
Dental fluorosis prevalence (> 1.5 mg/L water F)	Villages in Vadodara district, Gujarat, India, with > 1.5 mg/L F water	430	56.22	69.01	%	1.23	^62^	45–64	Adults
Dental fluorosis prevalence (> 1.5 mg/L water F)	Villages in Vadodara district, Gujarat, India, with < 1.5 mg/L F water	156	27.17	37.5	%	1.38	^62^	65 <	Adults
Dental fluorosis prevalence (> 1.5 mg/L water F)	Villages in Vadodara district, Gujarat, India, with > 1.5 mg/L F water	183	45.74	52.8	%	1.15	^62^	65 <	Adults
Dental fluorosis prevalence	Thailand	125	1.1	0.8	DMFT scores	0.73	^66^	,	Adolescents
Dental fluorosis prevalence (< 1.5 mg/L water F)	Villages in Vadodara district, Gujarat, India, with < 1.5 mg/L F water	487	44.15	45.49	%	1.03	^62^		Adults
Health risk	Vattamalaikarai River basin, South India	118	73	64	%	0.88	^76^		Adults

Past studies show that, under similar conditions of exposure, boys below puberty (up to 11 years of age) are likely to suffer stronger and more prevalent adverse effects of F overexposure than girls of the same age bracket. Likewise, the literature sources show greater potential of adolescent boys (12–18 year) to suffer more severe F impacts than the girls and this discrepancy appear to increase against the male populations in older cohorts than in the younger ones.

The literature data (Table 5) collaborated the results of the current study as well as those of similar studies on toxic trends of other environmental agents. Many studies assessing sex-related differences in human responses to toxic or pharmacologic agents have substantiated the existence of differential response between males and females^77^. In one study, the males exhibited a greater potential to suffer greater DNA-damage from exposure to DNA damaging agents^78^. Yet, in another study, the differential response to toxic heavy metals in men and women indicated that differences, such being reported, can emanate both from variation in the levels and structure of exposure to the deleterious agents and from differential physiological responses of the male and female bodies to the stimulus^79^.

The understanding of sex differences in susceptibility to toxic impacts of fluoride present information of considerable public health importance, which can have formidable socioecological and socioeconomic implications. Nonetheless, other than the age and sex of the subjects affecting the degree and impact of F exposure among the affected communities, the physical activity levels (PAL) and the body weight (BW) of the subjects appear to exert greater effect in influencing the levels and impacts of deleterious F exposure. The risk levels of F overexposure increased with increasing PAL and the effects were magnified by greater BW. This is because both the BW and PAL increase relative food consumption by individuals and heightens the F overexposure levels from the polluted foodstuffs. Thus, the high risk of F overexposure, and the prevalence of adverse F impacts, among male populations in the current study could linked to the greater average BW, and higher average PAL, among the males than among the corresponding female populations. Higher average BW, and PAL, cause the average males to consume greater quantities of food, and water, ingesting greater amounts of F from the F-contaminated diets and resulting in greater F exposure and toxicity.

Consequently, the combined influence of sex, age, PAL and BW on F exposure was greatest in young boys than in any other category of the populations.

## Conclusions

According to the findings of the current work, the following conclusions could be drawn:

1. The study revealed high F levels exceeding the RDA level of 4 mg/kg in potatoes (*Solanum tuberosum L.*), beans (*Phaseolus vulgaris L.*) and peas sampled from Nakuru County.
2. High F was recorded in the three foodstuffs from all the sub regions of the current study area but the F levels in both the beans and the potatoes varied from region to region showing that they were controlled by conditions that control F availability in the environment but the F levels of peas (*Possum sativa*) samples were lower and uniform across the regions showing that they were not controlled by regional variations in F levels in the environment.
3. The risk of F overexposure through the contaminated foodstuffs was greater in younger children than in adult populations, in male populations than in the female populations, and in individuals with higher BW and PAL. As a results, the study found that young boys could be at the greatest risk of fluoride toxicity than any other groups, especially if they also presented symptoms of above average BW.
4. Elevated levels of fluorides in peas, beans and potatoes have adverse public health implications (of risk of fluoride overexpose) to the resident communities through food, which calls for public awareness campaigns, especially among farmers, food producers and consumers as well as for the development and disseminate appropriate prevention and F amelioration strategies.

## Data Availability

Available by placing requests to Dr. Enos Wambu who is the Corresponding Author.

## Abbreviations

[F]: Fluoride concentration (mg/kg or mg/L)
AF: Absorption factor for foodstuffs
ANOVA: Analysis of variance
BMR: Basal metabolic rate
DDW: Doubly-deionized water
DEI: Daily energy intake through food (kcal/kg/day)
E: Dietary calorific value (kcal/kg) of food
EADD: Estimated average daily F dosage
EARS: Eastern African Rift Valley System
F: Fluoride
FEC: Percentage daily energy contribution of food
RDA: Recommended dietary allowance
TEE: Total energy expenditure in 24 h
TISAB: Total ionic strength adjustment buffer
